# Accumulation of Poly(3-hydroxybutyrate) Helps Bacterial Cells to Survive Freezing

**DOI:** 10.1371/journal.pone.0157778

**Published:** 2016-06-17

**Authors:** Stanislav Obruca, Petr Sedlacek, Vladislav Krzyzanek, Filip Mravec, Kamila Hrubanova, Ota Samek, Dan Kucera, Pavla Benesova, Ivana Marova

**Affiliations:** 1 Materials Research Centre, Faculty of Chemistry, Brno University of Technology, Purkynova 118, 612 00, Brno, Czech Republic; 2 Institute of Scientific Instruments, Academy of Sciences of The Czech Republic, Vvi, Kralovopolska 147, 612 64, Brno, Czech Republic; Tsinghua University, CHINA

## Abstract

Accumulation of polyhydroxybutyrate (PHB) seems to be a common metabolic strategy adopted by many bacteria to cope with cold environments. This work aimed at evaluating and understanding the cryoprotective effect of PHB. At first a monomer of PHB, 3-hydroxybutyrate, was identified as a potent cryoprotectant capable of protecting model enzyme (lipase), yeast (*Saccharomyces cerevisiae*) and bacterial cells (*Cupriavidus necator*) against the adverse effects of freezing-thawing cycles. Further, the viability of the frozen–thawed PHB accumulating strain of *C*. *necator* was compared to that of the PHB non-accumulating mutant. The presence of PHB granules in cells was revealed to be a significant advantage during freezing. This might be attributed to the higher intracellular level of 3-hydroxybutyrate in PHB accumulating cells (due to the action of parallel PHB synthesis and degradation, the so-called PHB cycle), but the cryoprotective effect of PHB granules seems to be more complex. Since intracellular PHB granules retain highly flexible properties even at extremely low temperatures (observed by cryo-SEM), it can be expected that PHB granules protect cells against injury from extracellular ice. Finally, thermal analysis indicates that PHB-containing cells exhibit a higher rate of transmembrane water transport, which protects cells against the formation of intracellular ice which usually has fatal consequences.

## Introduction

Microorganisms are exposed to a series of various stress factors in the environment, among which great temperature oscillations are very frequent. It should be noted that approximately 80% of our planet’s biosphere is permanently cold with average temperatures below 5°C and that even in the remaining regions the temperature fluctuates wildly, occasionally falling close to, or even below 0°C [1]. Therefore, many microorganisms have developed sophisticated strategies to help them endure low temperatures. These include the capacity to produce and accumulate cryoprotectants, substances which are able to protect cells from the adverse effects of freezing and low temperature [2]. There are numerous low molecular weight solutes (e.g. amino acids and their derivatives, sugars, ectoines and their derivatives, etc.) as well as high molecular weight substances (e.g. proteins and polysaccharides) which are produced by bacteria and exhibit cryoprotective properties [2, 3].

Low temperatures induce distinct responses among bacterial cells depending on the actual temperature. Exposure to cold conditions above 0°C is usually accompanied by an active response from bacterial cells. On the contrary, the response of most prokaryotes is passive at subzero temperatures, which is connected with the formation of ice [4]. As ice crystals grow, a process which occurs initially in the extracellular medium, the concentration of the solutes in the medium is excluded into an ever-decreasing solvent volume which results in effective osmotic stress. This induces so-called “freeze-dehydration” which is an important harmful consequence of cell freezing. Another important damaging mechanism identified during freezing of the cells is formation and propagation of intracellular ice. It is proposed that crystals of intracellular ice cause the physical destruction of membranes, formation of gas bubbles, and might also result in organelle disruption [3]. More generally, the survival of bacterial cells during freezing depends on the cooling rate. Cell survival is maximal when cooling occurs slowly enough to avoid formation of intracellular ice but fast enough to prevent causing injury to the cells by substantial dehydration [5]. Apart from dehydration and intracellular ice formation, cells can also be harmed by reactive oxygen species (ROS) formed in cells during freezing [6]. In addition, a decrease in the size of the unfrozen channels in ice during freezing can cause shrinkage of the cells, resulting in mechanical injury [7].

Polyhydroxyalkanoates (PHAs) are storage polymers accumulated in the form of intracellular granules by a wide range of taxonomically different groups of microorganisms. Among the wide variety of PHAs, the polyester of 3-hydroxybutyrate, poly(3-hydroxybutyrate) (PHB), is the most common and the best studied [8]. The biosynthesis and degradation of intracellular PHB occur in cells simultaneously, and therefore the metabolism of PHB exhibits a cyclic mechanism [9]. It is generally proposed that PHAs serve primarily as a carbon and energy storage material when exogenous carbon sources are depleted. However, there are reports that the capacity for intracellular PHA accumulation and degradation also enhances the resistance of bacterial cells to various stress conditions including low temperatures and freezing.

PHAs have been observed to be essential for maintenance of the redox state in the Antarctic bacterium *Pseudomonas* sp. 14–3 during low-temperature adaptation [10]. Iustman et al. isolated and studied the Antarctic strain *Pseudomonas extremaustralis*, whose high resistance to a wide range of stress conditions including cold was attributed to its capacity to produce PHB [11]. The PHB biosynthetic genes of the bacterium are located within an adaptive genomic island and were probably acquired by horizontal gene transfer, which suggests the importance of PHA accumulation in adaptation to stress conditions, such as those found in the extreme Antarctic environment [12]. Further, Pavez et al. reported that PHAs exert a protective effect against freezing in *Sphingopyxis chilensis* [13]. Numerous PHA-producing bacterial strains have also been isolated from Antarctic freshwater [14] and Antarctic soil [15], which indicates that PHA accumulation is a common metabolic strategy adopted by many bacteria to cope with cold environments.

Hence, in this work we investigated and assessed the potential protective mechanisms of PHB when bacterial cells are exposed to freezing and thawing. We have recently revealed the monomer of PHB– 3-hydroxybutyrate (3HB)–to be a very potent chemical chaperone capable of protecting model enzymes against heat-mediated denaturation and oxidative damage. Due to continuous PHB synthesis and degradation in bacterial cells (the so-called PHB cycle), the 3HB concentration in the PHB accumulating strain *Cupriavidus necator* was more than 16.5-fold higher than in the strain unable to accumulate PHA [16]. In this work, we tested the cryoprotective effect of 3HB for lipase as a model enzyme as well as for selected eukaryotic (S*accharomyces cerevisiae*) and prokaryotic (*Cupriavids necator*) microorganisms. In addition, using flow cytometry, thermal analysis, and Cryo-Scanning Electron Microscopy (Cryo-SEM), we tried to shed light on the complex role of intracellular PHB granules with respect to the survival of bacterial cells during freezing and thawing.

## Materials and Methods

### Materials and microorganisms

Trehalose, 3-hydroxybutyrate, p-nitrophenylpalmitate and lipase from *Rhizopusoryzea* were purchased from Sigma Aldrich, Germany. *Cupriavidus necator* H16 (CCM 3726) was obtained from the Czech Collection of Microorganisms, Brno, Czech Republic. The PHB non-producing strain *Cupriavidus necator* PHB^-4^ (DSM-541) was purchased from the Leibnitz Institute DSMZ-German Collection of Microorganism and Cell Cultures, Braunschweig, Germany. *Saccharomyces cerevisiae* CCY 21-4-47 was purchased from the Culture Collection of Yeast, Bratislava, Slovakia.

### Screening of cryoprotective effect of 3HB for lipase against freezing-thawing treatment

Samples of lipase (0.4 mg/ml) in 100 mM of phosphate buffer (pH 7.4) were prepared in the presence (50 and 100 mM) or absence of 3HB and in presence of 100 mM trehalose. The freezing-thawing stabilizing effect of 3HB on lipase was studied by incubating the samples (initial volume 1 ml) at -30°C for 2 h and thawing them at 30°C. After each cycle, aliquots (100 μl) for the determination of residual activity were taken.

The enzyme activity of lipase samples was determined spectrophotometrically according to the established procedure described by Pinsirodom and Parkin with a slight modification [17]. The assays were performed in standard 96-well microplates; the reaction mixture consisted of 230 μL of 100 mM phosphate buffer pH 7.4, 25 μL of 420 μM p-nitrophenylpalmitate substrate solution and 25 μL of suitable diluted enzyme solution. The reaction was started by the addition of substrate and the formation of the product (p-nitrophenol) at 40°C was followed at 405 nm using a Biotek ELx808 microplate reader. Under the specified conditions, 1 unit of enzyme activity was defined as the amount of enzyme releasing 1 μmol of the product per minute. All the analyses were performed in triplicates.

### Evaluation of the cryoprotective effect of 3HB for yeast and bacterial cells

The yeast culture *Saccharomyces cerevisiae* was cultivated (25°C, 140 rpm) in 250 mL Erlenmeyer flasks containing 100 mL of YPD medium (20 g/L glucose, 20 g/L peptone, 10 g/L yeast extract) for 24 hours. Then, 2 mL aliquots were taken, washed with PBS buffer, and re-suspended in PBS buffer in the absence or presence of 3HB (50 mM and 100 mM). The cell suspensions were frozen at -30°C for 2 h, then thawed at laboratory temperature and the viability of the yeast culture was analyzed immediately. We performed 5 subsequent freezing-thawing cycles, each sample prepared in triplicate. In a further experiment, we compared the cryoprotective effect of 100 mM of 3HB with trehalose and glycerol applied at the same concentration level. In this experiment, the yeast culture was adapted to the presence of cryoprotectants at 4°C for 1 h, after which freezing-thawing cycles were performed as described above.

Similar experiments were also performed with the PHB-producing bacterial strain *C*. *necator* H16 and its PHB non-accumulating mutant *C*. *necator* PHB^-4^. Erlenmeyer flasks (volume 250 mL) containing 100 mL of Mineral Salt (MS) medium (the MS medium was composed of 20 g fructose, 3 g (NH_4_)_2_SO_4_, 1 g KH_2_PO_4_, 11.1 g Na_2_HPO_4_· 12 H_2_O, 0.2 g MgSO_4_, 1 mL of microelement solution and 1 L of distilled water; the microelement solution was composed of 9.7 g FeCl_3_, 7.8 g CaCl_2_, 0.156 g CuSO_4_· 5 H_2_O, 0.119 g CoCl_2_, 0.118 g NiCl_2_ in 1 L of 0.1 M HCl) were inoculated with 5 mL of the overnight culture of the particular strain of *C*. *necator* grown in Nutrient Broth medium (NB medium: 10 g peptone, 10 g beef extract, 5 g NaCl in 1 L of distilled water). After 72 h of cultivation, 2 mL aliquots were taken, washed with PBS buffer, and re-suspended in PBS buffer in the absence or presence of 100 mM of 3HB. The cell suspensions were frozen at 30°C for 2 h, then thawed at laboratory temperature, and the viability of the bacterial culture was analyzed immediately. The freezing-thawing cycling was repeated 4 times, each sample prepared in triplicate. In addition, PHB content in the bacterial cultures was determined as described previously [18].

The capacity of *C*. *necator* H16 and *C*. *necator* PHB^-4^ to endure different freezing temperatures was also tested. Both bacterial strains were cultured as described above for 72 h, washed and re-suspended in PBS buffer, incubated at -5, -10, -15, and -20°C for 60 minutes, and thawed at laboratory temperature. After that, the viability of each bacterial culture was determined immediately.

The viability of yeast and bacterial cell populations was assessed by means of membrane integrity assay. Flow cytometry analysis using propidium iodide according to Coder was employed [19]. After each freezing-thawing cycle, 100 μl aliquots were taken, washed with PBS buffer, and diluted to a cell count of approx. 5·10^6^ per ml. Then, 1 ml of cell suspension was stained by 1 μl of 1 mg/ml propidium iodide in the dark for 5 min. After that, the viability of the cells was analyzed by flow cytometry (Apogee A50, Apogee, GB) using a 488 nm laser for the excitation and the red channel (FL3) for the fluorescence detection.

### Thermal analysis of bacterial cells

Basic calorimetric and thermogravimetric assays of bacterial samples were used in order to estimate whether the presence of PHB granules in cells can somehow affect the activity of the cellular water. For this purpose, we used bacterial cultures of *C*. *necator* H16 cultivated for 72 h in MS and NB medium, respectively. The fact that the strain does not accumulate PHB in NB medium is well documented [20] and was experimentally confirmed by the determination of the total PHB content in the cell by GC-FID as described previously [18]. Samples for the thermal analyses were always washed carefully with deionized water and centrifuged and the excess water was removed.

Differential scanning calorimetry was performed using a temperature-modulated calorimeter (DSC Q2000, TA Instruments, DE) equipped with an RCS90 cooling accessory and assessed by TA Universal Analysis 2000 software. All experiments were performed in hermetically sealed Tzero aluminum pans under a dynamic nitrogen atmosphere. Temperature-modulated DSC in the standard and quasi-isothermal modes, respectively, were applied in order to investigate the thermal effects of the freezing-thawing behavior of the bacterial cultures. In the former technique (MTDSC), the sample is cooled substantially from 20°C to -50°C and then heated back up with an underlying cooling/heating rate of 5°C/min and a temperature modulation of ± 1°C every 60 s. Quasi-isothermal temperature-modulated DSC (QiMTDSC; for details, see e.g. Otun et al. [21]) is a variant of traditional temperature-modulated DSC which involves the holding and modulation of a sample at a specific temperature for extended periods of time. This temperature can be increased or decreased incrementally in the course of the experiment. In the performed QiMTDSC experiment, samples were cooled down from 5°C to -30°C and heated back up in 1°C increments with an isotherm of 10 min at each increment. A temperature modulation of ±1°C every 60 s was applied.

Thermogravimetry (TGA, TA Instruments, Q5000IR) was used to determine the weight loss in the temperature interval 25–700°C under a dynamic dry air atmosphere with a heating rate of 10°C/min. Weight loss in the interval 25–200°C was used to calculate the total water content in the samples. In addition, an isothermal TGA experiment at 60°C was performed in order to provide further comparison of the dynamics of the drying process for the samples under investigation.

### Cryo-SEM

Bacterial cultures of *C*. *necator* H16 or *C*. *necator* PHB^-4^ grown on Petri dishes with the mineral medium described above were collected from agar plates, quickly frozen in liquid nitrogen and moved into a cryo-vacuum chamber (ACE600, Leica Microsystems), where they were freeze-fractured and briefly sublimated at -95°C. Further, the samples were moved at high vacuum using a shuttle (VCT100, Leica Microsystems) into a Scanning Electron Microscope (Magellan 400/L, FEI) equipped with a cold stage and the fractured structures were observed in a 1 keV electron beam at -135°C without any metal coating.

## Results

### Cryoprotective effect of 3HB for lipase

**Fig 1:**
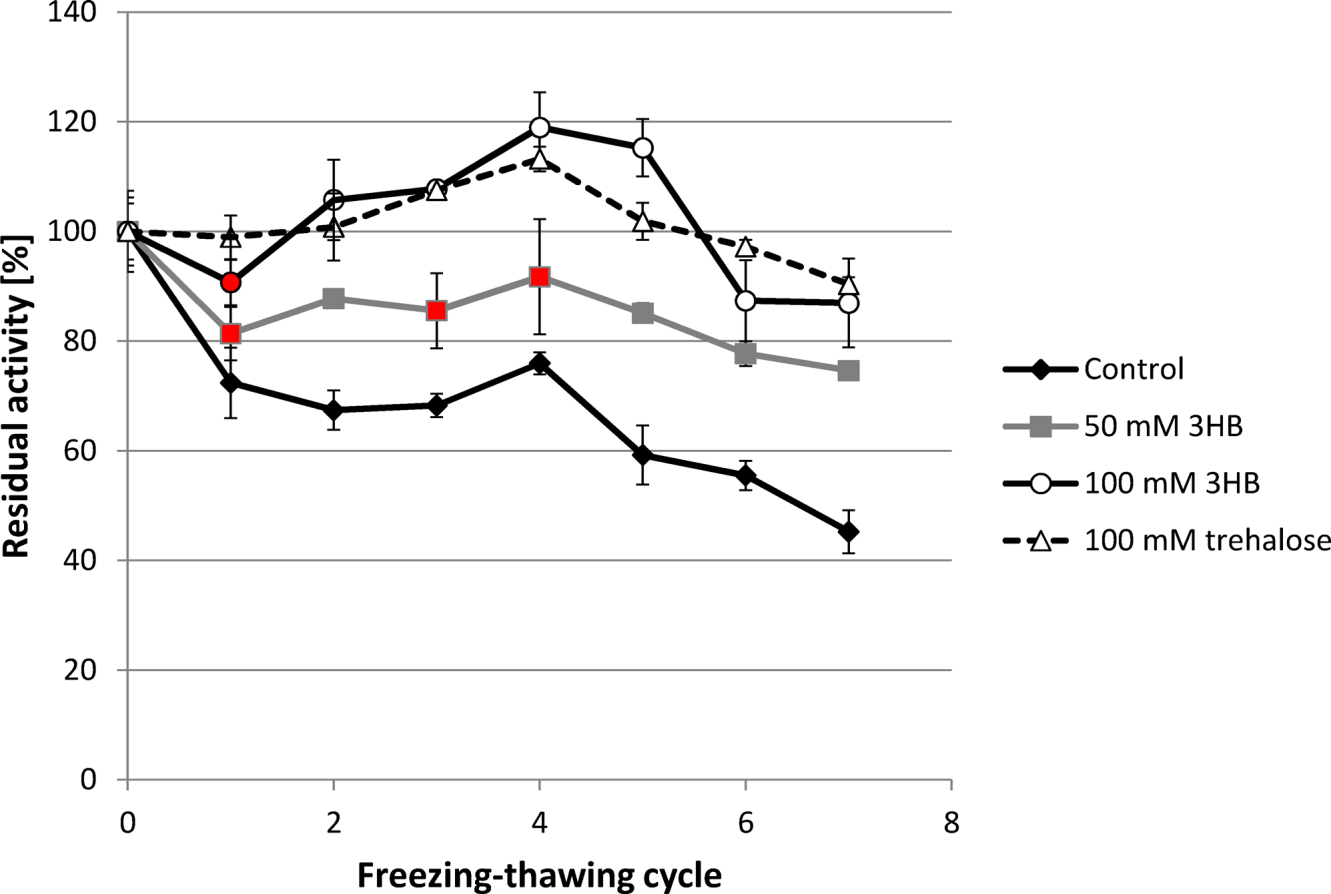
Effect of freezing-thawing treatment on the residual activity of lipase in the absence and presence of 50 mM and 100 mM 3HB. *note: Statistical significance was tested using 2 sample t-test (Minitab), each sample was compared with control, statistically insignificant results are labeled by red.

In our previous work, we observed that 3HB serves as a very effective protectant of enzymes (in particular, lipase from *Rhizopus oryzea* and lysozyme) against denaturation caused by high temperature and oxidative damage [16]. Hence, we also tested its cryoprotective efficiency using lipase–the same model enzyme. The enzyme was solubilized in phosphate buffer in the absence and presence of 3HB, which was tested at two concentration levels: 50 and 100 mM. Further, the enzyme was also tested in presence of well-established cryoprotectant trehalose, which was applied at 100 mM. The samples were subjected to repeated freezing-thawing cycles and the activity of the enzyme was determined after each cycle (Fig 1). The freezing-thawing treatment significantly reduced the activity of the enzyme in the control sample (without 3HB) which, after the 7th cycle, exhibited only 45.23% of its initial activity. In contrast, 3HB exhibited a significant concentration-dependent protective effect, since the relative enzyme activity was always higher when 3HB was present in the sample. The higher the dose of 3HB, the higher the enzyme activity that was observed. Surprisingly, when 100 mM of 3HB was applied, the relative enzyme activity of the enzyme in the sample actually increased during the initial 5 freezing-thawing cycles and a significant decrease in the activity was not observed until the 6th cycle; even after the 7th cycle the enzyme still possessed 87% of its relative activity. Protective effect of 100 mM 3HB is comparable to that of 100 mM trehalose, application of which also surprisingly increased enzyme activity during initial freezing-thawing cycles and after the 7th cycle 90% of its initial activity was recorded.

An increase in enzyme activity after freezing in presence of cryoprotectants was observed also by other authors and is ascribed to favorable conformation changes (partial unfolding) of enzyme at the level of active site and/or its neighborhood which leads to a higher affinity between enzyme and substrate [22, 23].

### 3HB as a protectant of *Saccharomyces cerevisiae*

**Fig 2:**
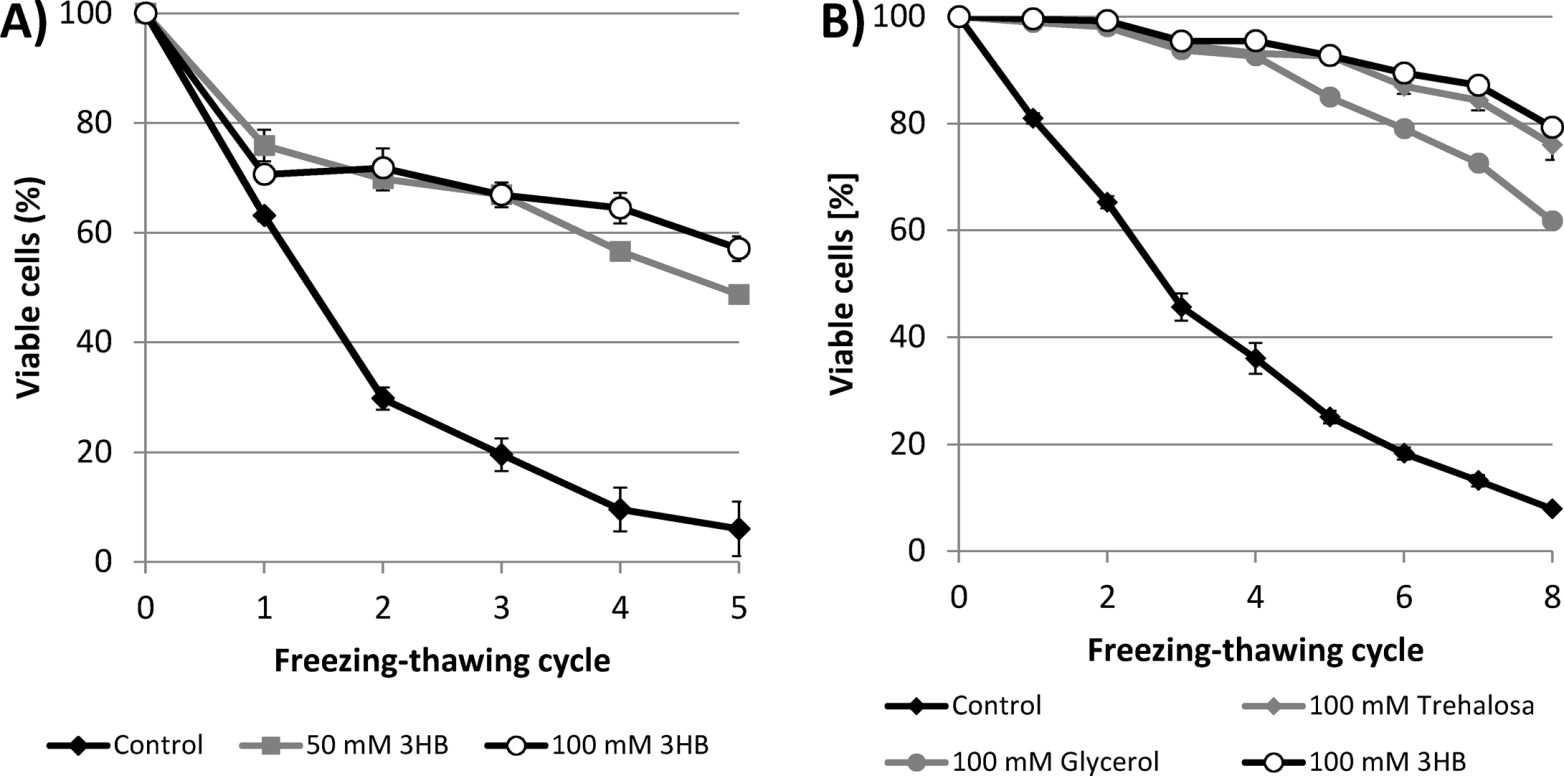
**A) Viability of *S*. *cerevisiae* culture during freezing-thawing challenge in the presence of 3HB applied at 50 mM and 100 mM and in its absence B) Viability of the yeast culture exposed to repeated freezing-thawing in the presence of 3HB, trehalose and glycerol (all protectants were applied at 100 mM concentration) and in absence of a cryoprotectant.** *note: Statistical significance was tested using 2 sample t-test (Minitab), each sample was compared with control, all the differences were found statistically significant.

In the first experiment, we demonstrated the cryoprotective activity of 3HB for enzymes. Therefore, we decided to test its ability also to protect whole cells against damage induced by freezing-thawing cycles. First, we utilized the yeast *Saccharomyces cerevisiae* as a model eukaryotic microorganism. Yeast cells suspended in PBS buffer with and without 3HB (50 and 100 mM) were repeatedly frozen at -30°C and thawed and the viability of the yeast culture was assayed after each cycle. Also in this case, 3HB exhibited a significant cryoprotective effect: the viability of the yeast culture was always higher in the presence of 3HB than in the control culture (Fig 2A). After 5 freezing-thawing cycles, only 6% of cells in the control culture were viable, whereas the viabilities of the cultures with 50 and 100 mM 3HB were 48.7 and 57.1%, respectively.

In a further experiment, the cryoprotective effect of 3HB was compared with that of two other well-established cryoprotectants–trehalose and glycerol [3]. All the tested solutes were applied at a concentration of 100 mM. Unlike in the previous experiment, before exposure to the freezing-thawing cycles, the yeast culture was left to adapt to the cryoprotectants at 4°C for 1 h (the control culture was treated in the same manner). Such adaptation was reported to increase the effectiveness of most cryoprotectants [2]. A comparison of the protective effects of the tested solutes is presented in Fig 2B. All the tested solutes significantly enhanced the viability of the yeast culture in comparison with the control culture. After the 8th cycle, only 7.9% of cells in the control sample were viable, whereas the viability of yeast culture challenged in the presence of cryoprotectants did not fall below 60%. Among the tested cryoprotectants, 3HB exhibited the greatest protective effect, which was slightly greater than that of trehalose (the proportion of viable cells in the yeast culture after the 8th cycle was 79.3 and 76.0% in 3HB and trehalose, respectively). The protective effect of glycerol was less pronounced, with 61.7% of cells remaining viable after 8 freezing-thawing cycles. Hence, 3HB seems to be a very effective cryoprotectant, even in comparison with trehalose and glycerol, which are considered to be natural cryoprotectants used by various microbes and are also routinely applied in the cryopreservation of microorganisms [2; 24].

### Cryoprotective effect of 3HB and PHB for *Cupriavidus necator*

**Fig 3:**
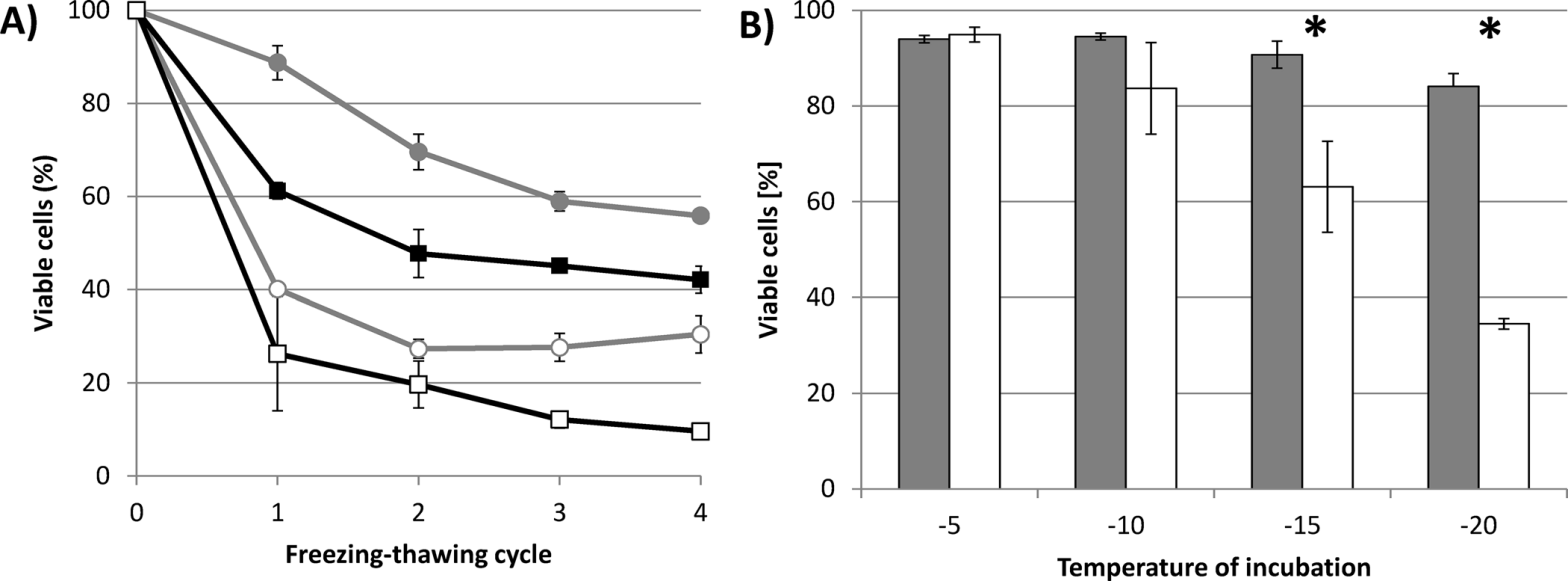
**A) Viability of PHB-accumulating *C*. *necator* H16 and PHB non-accumulating *C*. *necator* PHB**^**-4**^ **exposed to repeated freezing-thawing challenge in the presence and absence of 100 mM 3HB. B) Viability of *C*. *necator* H16 and *C*. *necator* PHB**^**-4**^ **exposed to various sub-zero temperatures without addition of 3HB.** *note: Statistical significance was tested using 2 sample t-test (Minitab). Presence of 3HB resulted in statistically significant increase in viability for both tested cultures and for all freezing-thawing cycles. Difference between viability of *C*. *necator* H16 and *C*. *necator* PHB–4 was statistically significant for all experimental conditions except for the initial 2 freezing-thawing cycles in absence of 3HB. In Fig 3B viability of *C*. *necator* H16 was compared with viability of *C*. *necator* PHB^-4^ for each temperature of incubation; statistically significant differences are labeled by asterisks.

The protective effect of 3HB during freezing-thawing cycles was also tested with two bacterial cultures: PHB-producing *Cupriavidus necator* H16 and its mutant strain *Cupriavidus necator* PHB^-4^, which, due to a mutation of the gene encoding for PHB synthase, is not capable of accumulating PHB [25]. The bacterial cultures were cultivated for 72 h, at which point both cultures had reached the stationary phase of their growth. The PHB content in *C*. *necator* H16, determined by GC-FID, reached 76% of cell dry weight; the PHB content in the culture of *C*. *necator* PHB^-4^ was negligible (about 0.4% of cell dry weight). Both cultures were exposed to freezing-thawing cycles (the temperature of freezing was -30°C) suspended in PBS buffer in the presence or absence of 100 mM of 3HB. The results are shown in Fig 3A.

Similarly to the previous experiments with *S*. *cerevisiae*, the application of 3HB significantly improved the survival of both bacterial cultures, as compared to samples without 3HB. Moreover, we observed a significantly higher proportion of viable cells in the bacterial culture capable of accumulating PHB than in the PHB non-accumulating mutant in both experimental settings–with and without 3HB. In addition, bacterial cultures in the absence of 3HB revealed a different profile to their viability curves during the freezing-thawing challenge compared to yeast cultures or bacterial cultures with 3HB. In the yeast cultures and also in the bacterial cultures with 3HB, viability decreased more or less constantly during the whole freezing-thawing test. In contrast, without exogenous 3HB, most bacterial cells lost their viability during the first freezing-thawing cycle (the proportions of viable cells after the 1st cycle were 40.1 and 26.3% in *C*. *necator* H16 and *C*. *necator* PHB^-4^, respectively). After that the viability of the cultures remained constant or decreased only slowly.

Therefore, we decided to compare the resistance of both bacterial strains without the addition of 3HB during a single freezing-thawing cycle challenge in which different temperatures of freezing (-5, -10, -15, and -20°C) were applied. The bacterial strain capable of PHB accumulation revealed a significantly higher ability to endure freezing than its PHB non-producing mutant strain. With decreasing temperature the differences between the viabilities of the bacterial strains increased (see Fig 3B). When the bacterial strains were exposed to -5°C, the proportions of viable cells in *C*. *necator* H16 and *C*. *necator* PHB^-4^ were 94.0 and 94.9%, respectively. However, at -20°C, in the culture of *C*. *necator* H16 84.1% of cells retained viability, while only 34.5% of cells were identified as viable in *C*. *necator* PHB^-4^. Hence, it seems that the presence of PHB granules in cell cytoplasm represents a significant advantage when the cells are exposed to subzero temperatures: the lower the temperature, the more pronounced the protective effect that was observed.

In addition, to investigate whether PHB granules are actively metabolized by bacterial culture *C*. *necator* H16 during repeated freezing-thawing, we performed experiment in which we determined PHB content in the bacterial culture after the each cycle; data are provided in S1 Fig. Since none decrease in the PHB content was observed, it can be stated that the bacterial culture did not utilize PHB granules to increase intracellular level of 3HB and it seems that in perspective of PHB metabolism response of the bacterial culture to repeated freezing is rather passive than active.

### Observation of PHB granules by Cryo-SEM

**Fig 4:**
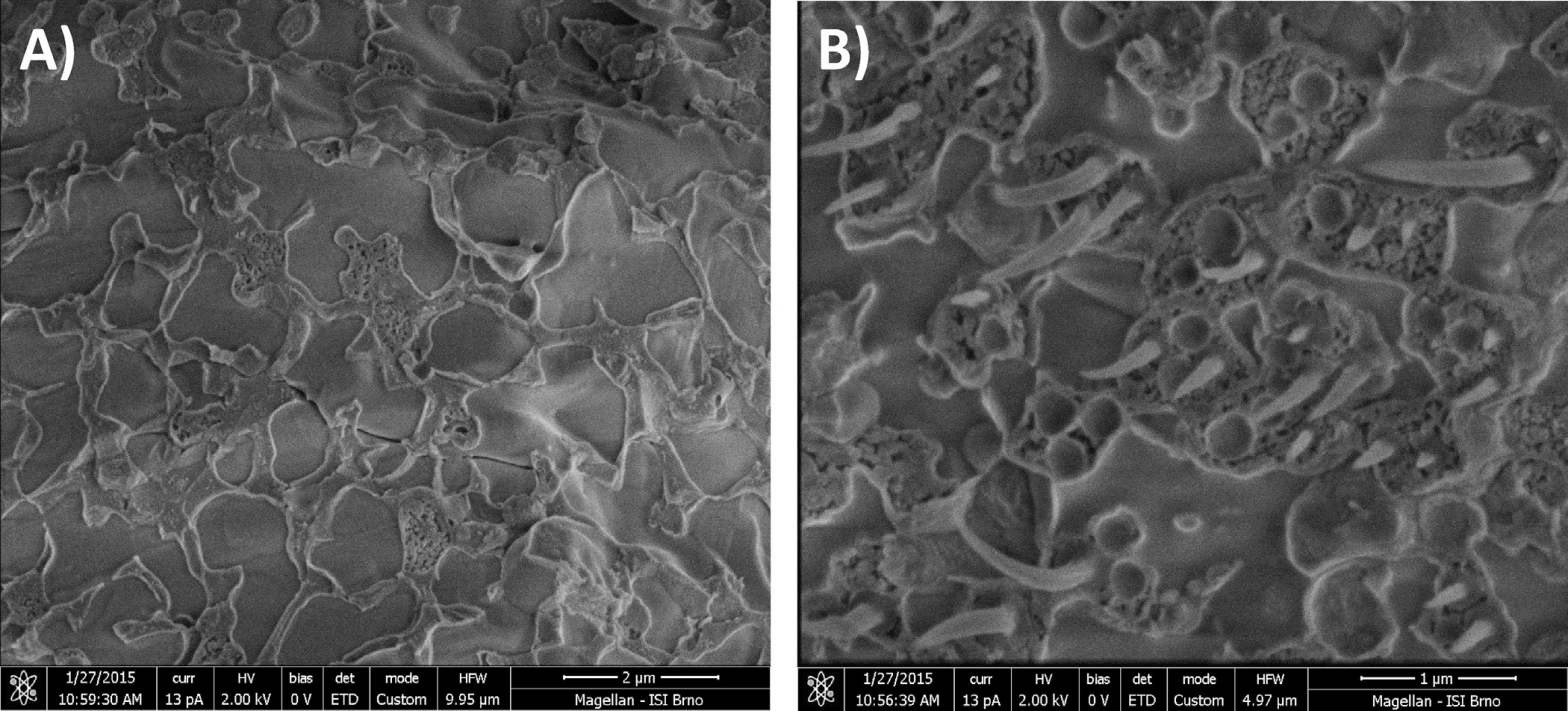
Cryo-SEM microphotographs of A) PHB non-producing *C*. *necator* PHB^-4^, B) PHB-accumulating cells of *C*. *necator* H16.

Cryo-SEM is a very interesting technique providing an ultrastructural insight into various biological samples in a deeply-frozen state. Therefore, we used this method to investigate the morphology of frozen intracellular PHB granules. Fig 4 shows Cryo-SEM microphotographs of PHB non-containing (*C*. *necator* PHB^-4^) and containing (*C*. *necator* H16) bacterial cells. In PHB-containing cells, needle-like plastic deformations were observed, while these structures were absent in cells without polymer, which indicates that these deformations can be clearly attributed to PHB granules. Employing the same experimental approach, similar structures were also observed by [26] in the PHA-producing bacteria *Comamonas acidovorans*. Despite the fact that the mechanism of the genesis of these deformations during freeze-fraction has not yet been explained, we can state that frozen PHB granules exhibit completely different mechanical and physico-chemical properties than any other components of bacterial cytoplasm and that their flexibility, even in deeply-frozen states, is significantly higher than that of PHB isolated from bacterial cells. When PHB polymer is extracted from cells, its elongation-to-break is about 4% [8], while in Cryo-SEM microphotographs we observed elongation corresponding to a value of more than 100%.

### Effect of PHB on the freezing/melting of cellular water studied by DSC

**Fig 5:**
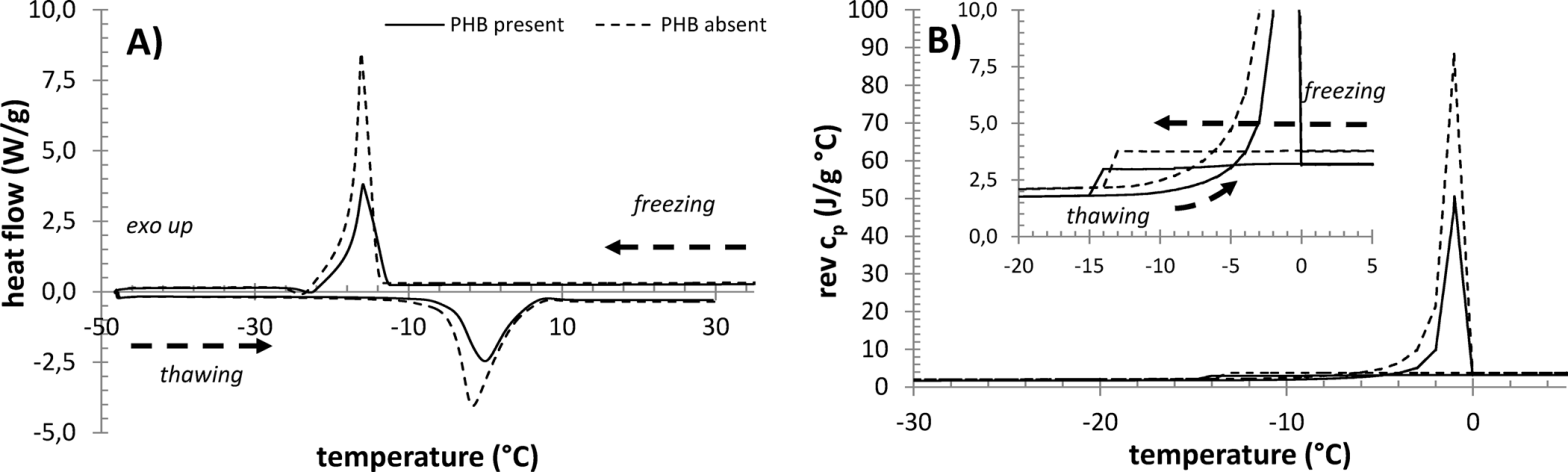
Results of DSC analysis of centrifuged PHB-containing and PHB non-containing cultures of *C*. *necator*. A) MTDSC thermograms, B) QiMTDSC thermograms.

A comparison of standard MTDSC thermograms recorded for *C*. *necator* H16 samples cultivated in the two different media is shown in Fig 5A. The freezing and thawing of water in the samples are represented by corresponding exotherms and endotherms, respectively. The shape and position of the freezing exotherm provide little information about the state of water in the system, because the freezing phenomenon is greatly affected by the inevitable and hardly reproducible effect of water supercooling. On the other hand, the endothermic signal, which corresponds to the melting of the frozen water, gives an interesting overview of the activity of water in the sample [27].

Two interesting observations can be derived from the thermograms shown in Fig 5A. First, the melting endotherm which corresponds to the culture sample containing PHB granules is shifted to higher temperatures. Second, the total area of the endotherm (which represents the weight-specific heat consumed during the water melting) is significantly lower. The latter feature is easily explainable by the fact that the relative water content in PHB-containing cells is naturally lower, which correspondingly decreases the area of the ice-melting endotherm. The specific heats of fusion of water in the samples, which were calculated from the total area of the melting endotherm and from the total water content in the sample determined by TGA analysis (see below), did not show any significant differences either between the two analyzed samples or in comparison with the heat of fusion of pure water. This indicates that neither of the samples contains any significant amount of water which does not freeze (often referred to as non-freezing water).

On the other hand, the shift of the ice-melting endotherm towards higher temperatures for the MS medium-cultivated culture represents a significant experimental finding. It was demonstrated that the shift is reproducible–it was found repeatedly, no matter which particular DSC protocol was applied. In all these experiments, not only the peak but also the onset point of the endotherm was always shifted. The particular magnitude of the shift depended on the experimental conditions, e.g. the heating rate. For the results shown in Fig 5A, the peak of the endotherm was shifted by approx. 1.5°C.

An invaluable feature of MTDSC analysis is that it allows separation of the heat-flow signals related to reversible and non-reversible processes, respectively. The deconvolution of the total heat-flow signal, presented in Fig 5A, is shown in S2 Fig. The results of this deconvolution confirm that the freezing of water in the samples is an almost completely non-reversible process that is caused by the above-mentioned supercooling of liquid water. On the other hand, a further difference in the water-melting process between the PHB-containing and non-containing cultures was revealed: the non-reversible component of the melting signal in the absence of PHB was significantly more pronounced.

### Effect of PHB on the drying of cells studied by TGA

**Fig 6:**
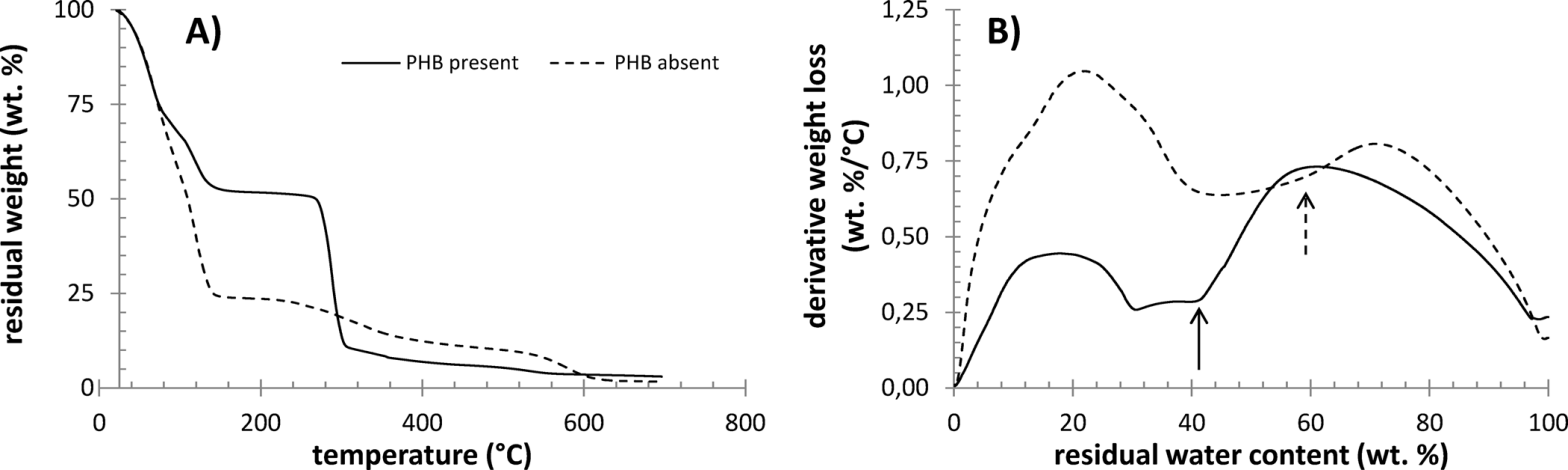
Results of dynamic TGA analysis of centrifuged PHB-containing and PHB non-containing cultures of *C*. *necator*. A) Weight loss at a heating rate of 10°C/min in the interval 25–700°C, B) derivative weight loss as a function of residual water content (arrows indicate critical water content).

TGA analysis was utilized in this study to provide further information on the activity of water in the studied bacterial cultures. Fig 6A shows the complete thermograms of PHB-containing and non-containing cells. The presence of PHB in the culture is evident from the significant weight loss at about 300°C, where the labile organic components of the cells (including PHB) are degraded. Additionally, it can be seen that the weight loss step associated with the drying of the sample is completed below 200°C. The total relative content of water, determined from the weight loss in this temperature interval, naturally differed for the two compared bacterial cultures. Nevertheless, when the content of PHB is excluded and the water content is given relative to the residual dry content of the cell, no important difference between the two samples is found. This shows that the presence of PHB granules does not significantly affect the total content of intracellular water.

Nevertheless, it has been proposed by several authors (e.g. by Uribelarrea et al. [28]) that TGA can be used also for the determination of different forms of water in cell samples. For this purpose, the derivation of water loss with temperature or time (i.e. the rate of drying of the sample) is given as a function of residual water content in the sample, and the critical points of this dependence are taken to be the indicators of moments at which a change in the mechanism of the drying process occurs. It can be seen in Fig 6B that these points can also be found for the tested samples. The arrows in the figure indicate the first critical point of the respective drying curve, when the release of the least strongly bound water is completed.

## Discussion

In our previous study we identified 3HB as a very potent chemical chaperone capable of protecting lipase and lysozyme against adverse effects of heat and oxidative damage [16]. Hence, we decided also to test its cryoprotective efficiency for enzymes as well as whole microbial cells. Cryoprotective ability has been observed for a wide spectrum of compatible solutes which are produced and accumulated by various microorganisms to cope with ubiquitous environmental stresses such as high osmolality or temperature fluctuations [29, 30]. The cryoprotective ability of these molecules is not only scientifically interesting from a general stress-response point of view, it also has potential practical applications since proteins and other biological molecules as well as whole cells are routinely preserved in a frozen state. Hence, the application of appropriate cryoprotectants can help to maintain the activity and/or viability of preserved biological samples [2, 31].

Freezing-thawing represents a complex combination of several stress factors which reduce the activity of enzymes. Apart from low temperature and the formation of ice crystals, proteins can also be damaged by increasing concentrations of buffer salts and co-solutes which is accompanied by pH changes and increase of ionic strength [32]. Compatible solutes are usually classified as kosmotropes, which means that interactions of a compatible solute and water are stronger than water-water interaction. This effect on the water network and the ordering of water molecules by compatible solutes, therefore, affect the process of ice formation, thus providing their cryoprotective effect [24, 30]. Furthermore, compatible solutes are capable of stabilizing enzymes by affecting their hydration shell, which might provide protection not only against the adverse effects of low temperature, but also the effects of high osmolality and pH changes, as was reported for ectoines and trehalose by Van Thouc et al. [33].

Although 3HB is usually not ascribed as a typical compatible solute, it seems to be a very efficient cryoprotectant of our model enzyme–lipase from *Rhizopus oryzea* (Fig 1). When 3HB was added at a 100 mM concentration, it was capable of completely protecting lipase against freezing-thawing mediated damage during the initial 5 freezing-thawing cycles, while in non-protected lipase we observed a decrease in relative activity to 59% of its initial value. Soto et al., reported that 3HB exhibited chemical chaperone activity preventing the aggregation of proteins of *Pseudomonas* sp. CT13 under combined salt and thermal stress [34]. However, to our knowledge, despite the fact that its cryoprotective efficiency is obvious, there are no reports regarding the cryoprotective activity of 3HB.

In comparison with enzymes, the spectrum of potential stresses associated with freezing of the whole cells becomes even wider. During freezing, cells experience either cellular dehydration (as a result of extracellular ice formation and consequent water transport driven by the resulting osmolality difference) or intracellular ice formation (IIF). These factors are oppositely dependent on the cooling rate during freezing (slow cooling leads to dehydration and fast cooling to IIF). While IIF is practically always lethal (cells have a finite volume; the expansion of water during freezing leads to rupture of the cell membrane), dehydration induces different cellular responses according to the particular freezing conditions–above all, the cooling rate [35]. Consequently, extensive cell dehydration causes freezing injury due to exposure to high concentrations of solute, while, on the other hand, partial cell dehydration protects the cell from IIF and is therefore used as a crucial step in cryopreservation protocols [5]. Obviously, the dynamics of the freezing process and of the cell response are crucial for cell survival.

Because of the complex nature of cell freezing, effective cryoprotectants should undertake multiple protective actions [2]. Our results show that 3HB served as a very potent cryoprotectant for *S*. *cerevisiae* cells (Fig 2); its protective efficiency was comparable with that of glycerol and trehalose, which are routinely used for the cryopreservation of microorganisms. Furthermore, 3HB exhibited even slightly higher cryoprotective activity than trehalose, itself suggested by Jain and Roy to be one of the best cryoprotectants known [24].

Apart from the mechanisms mentioned above, reactive oxygen species (ROS), generated as a consequence of an impaired aerobic respiration chain, also significantly contribute to the injury of cells during the freezing-thawing process [6]. In our previous study we observed that 3HB is, similarly to other compatible solutes such as ectoines [36], capable of protecting model enzymes from oxidative damage cause by hydrogen peroxide or heavy metals [16]. Hence, this important feature can also contribute to the cryoprotective activity of 3HB in yeast cells.

In a further experiment, we tested the cryoprotective potential of 3HB using two bacterial strains, PHB-producing *C*. *necator* H16 and its PHB non-accumulating mutant strain *C*. *necator* PHB^-4^. Similarly as in the yeast culture, and also in both bacterial cultures, the addition of 100 mM 3HB increased the proportion of non-damaged cells in freezing-thawing tests compared with cultures challenged in the absence of 3HB. This observation demonstrates the effectiveness of 3HB as a cryoprotectant of whole microbial cells and shows that 3HB also works in bacterial cells.

However, we also observed a significant difference in the viability of the tested bacterial cultures in the absence of exogenous 3HB. The PHB-producing strain *C*. *necator* H16 exhibited superior viability to its PHB non-accumulating strain during the entire freezing-thawing test. Thus, it seems that intracellular reserves of PHB also play an important role in the freezing survival of bacteria, which is in agreement with the observations of other authors. Pavez et al. identified PHA accumulation in the bacterium *Sphingopyxis chilensis* (isolated from an oligotrophic aquatic environment where the temperature oscillates around 0°C) as the most important feature protecting cells from freezing conditions [13]. Moreover, PHA-producing bacteria were isolated from Antarctic freshwater [14] and Antarctic soil [15] confirming PHA accumulation as an efficient adaptation strategy for avoiding damage produced by intracellular ice crystals, oxygen-reactive species, and severe dehydration. When exposed to low temperatures, PHAs were observed to be essential for maintenance of the redox state in the Antarctic bacterium *Pseudomonas* sp. 14–3 [10]. In addition, there are several reports describing the connection between PHA metabolism and cellular alternative sigma factor RpoS stimulating the expression of general stress response-associated genes [11, 37, 38]. Recently, Mezzina et al. reported that phasin, PHA granules associated protein of *Azotobacter* sp. FA-8, revealed chaperone-like activity in-vivo as well as in vitro [39]. However, in our opinion, the cryoprotective mechanism of PHAs is even more complex and is not yet completely understood.

Previously we reported that PHB-accumulating bacterial strain *C*. *necator* H16 contains a 16.5-fold higher intracellular level of 3HB than its PHB non-accumulating mutant. We estimated the intracellular concentration of 3HB in PHB-accumulating strains to exceed 100 mM [16]. Considering the cryoprotective capacity of 3HB, a complete and functional PHB cycle might be a very important factor providing a naturally higher level of cryoprotectant in cytoplasm. This might substantially contribute to the greater freezing survival of PHB-producing bacterial strains. Nonetheless, it seems that *C*. *necator* H16 does not actively hydrolyze PHB granules (S1 Fig) to enhance intracellular concentration of 3HB when exposed to repeated freezing, which is in agreement with general expectation that the response of microbial cells to subzero temperatures is usually passive [4].

It is likely that the involvement of PHB in subzero temperature survival is much more complex still. Goh et al. observed that *E*. *coli* cells harboring PHA biosynthetic genes of *C*. *necator* but unable to mobilize PHAs exhibited higher stress resistance to oxidative stress [40]. This indicates that only the presence of intracellular PHA granules significantly changes the properties of bacterial cells and influences their stress survival.

Bonthrone et al. reported that intracellular native PHA granules do not comprise rigid, non-flexible, highly crystalized polymer, but are rather formed by highly mobile amorphous elastomer, which is reminiscent of supercooled liquid in terms of its properties [41]. This is in agreement with the extremely flexible behavior of PHB observed by Cryo-SEM (Fig 5). Despite the fact that cells of *C*. *necator* H16 were fractured at very low temperature (-140°C), we observed pull-out structures corresponding to the elongation of PHB granules of more than 100%. It should be noted that PHB isolated from bacterial cells rapidly crystallizes and its elongation-to-break is about 4% [8]. Hence, intracellular native PHB granules exhibit unique properties which might also provide physical protection of cells against the formation of ice crystals and shearing-stress associated with the freezing of extracellular water. Mazur et al. stated that expanding ice fields during extracellular water freezing results in a decrease in the sizes of unfrozen channels, which may cause shrinkage, deformation, and injury of the cells. PHB granules might represent a highly flexible scaffold protecting bacterial cells from such harm [7].

Our thermal analytical study of bacterial cells also provides some indications of the influence of PHB granules on the state of intracellular water. The shift in the melting endotherm in the MTDSC thermogram to higher temperature, which was observed for the culture containing PHB granules, cannot be revealed solely on the basis of the results of thermal analysis. Nevertheless, several suggestions can be made. One possible explanation is that this effect is related to an alteration of the adhesive forces between water and cellular components in the presence and absence of PHB. The stronger the adhesive force, the more the water molecules are “pulled out” from the ice crystals and the lower the temperature at which the ice melts. Therefore, it can be hypothesized that the “dilution” of the strongly hydrophilic species in the intracellular space by the less hydrophilic surfaces of PHB granules can partially lower the strength of the hydration of the remaining solutes. Deconvolution of the MTDSC signal leads to the similar conclusion. Stronger non-reversible component of the melting signal in the absence of PHB supports the assumption that the released water is more strongly attracted to the cellular components in these cultures than in PHB-containing bacteria.

**Fig 7:**
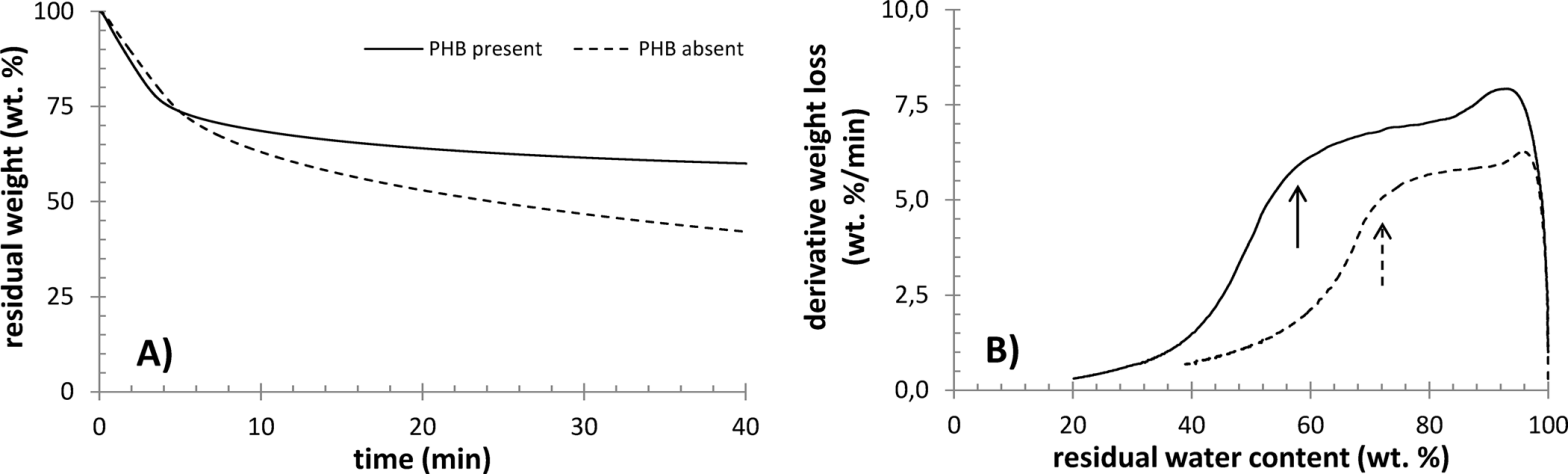
Results of isothermal TGA analysis of centrifuged PHB containing and PHB non-containing cultures of *C*. *necator*. A) Weight loss at 60°C, B) derivative weight loss as a function of residual water content (arrows indicate critical water content).

Also the thermogravimetric analysis of the cell drying experiments revealed similar differences in the state of water between the PHB accumulating and non-accumulation cultures. It is evident from the critical points shown in Fig 6B that the relative content of the least strongly bound form of water is higher in PHB-containing bacteria. The same conclusion can be drawn from the results of the isothermal drying experiment, which are presented in Fig 7. Comparison of Figs 6B and 7B indicates that the content of this form of water in the sample depends on the experimental conditions under which the drying occurred. This idea supports the assumption that this drying step is related to water which undergoes a kind of dynamic process such as transport through the cell membrane, desorption from the cell surface, etc.

In sum, the results of calorimetric and thermogravimetric analyses indicate that PHB-containing cells include water which can be more freely released from the cell either during drying or freezing. As discussed above, cell dehydration has diverse effects on cell survival according to the particular freezing conditions. When the cell faces severe dehydration, harmful solute effects cause the cell injury. On the other hand, when water release from the cell is completely suppressed, intracellular ice formation is not prevented and cell damage results from the rupture of the cell membrane. It is well documented that when the influence of the cooling rate is studied, the typical “inverted U” survival curve is obtained for a cell culture [7]. Obviously, a complex balance between the dynamics of external freezing stimuli (represented, for instance, by the rate of cooling) and cell response (the rate of dehydration) is needed in order to minimize the resulting cell mortality. Consequently, the effect of the presence of PHB granules on the state of water inside the cell and on the rate of its transmembrane transport may represent an important contribution to the overall cryoprotective strategy of PHB-producing bacteria.

## Supporting Information

S1 Fig Determination of PHB content (expressed as % of cell dry weight (CDW)) in *C*. *necator* H16 during freezing-thawing treatment (-30°C). (DOCX)

S2 Fig Results of the deconvolution of total MTDSC signals from Fig 5A into reversible (A) and non-reversible (B) components.
(DOCX)
