# Supplementary material for: Accumulation of Poly(3-hydroxybutyrate) Helps Bacterial Cells to Survive Freezing
Source: PLoS One. 2016 Jun 17;11(6):e0157778. doi: 10.1371/journal.pone.0157778 (PMC4912086; doi:10.1371/journal.pone.0157778)

**Accumulation of poly(3-hydroxybutyrate) helps bacterial cells to survive freezing**

Stanislav Obruca^1*a^, Petr Sedlacek^1a^, Vladislav Krzyzanek^2^, Filip Mravec^1^, Kamila Hrubanova^2^, Ota Samek^2^, Dan Kucera^1^, Pavla Benesova^1^, Ivana Marova^1^

*^1^Materials Research Centre, Faculty of Chemistry, Brno University of Technology, Purkynova 118, 612 00 Brno, Czech Republic*

*^2^Institute of Scientific Instruments, Academy of Sciences of The Czech Republic, Vvi, Kralovopolska 147, 612 64 Brno, Czech Republic*

*^a^Authors contributed equally*

**Corresponding author: Stanislav Obruca, Materials Research Centre, Faculty of Chemistry, Brno University of Technology, Purkynova 118, 612 00 Brno, Czech Republic. E-mail: Stana.O@seznam.cz, Tel: +420 541 149 486, Fax: +420 541 211 697*

**S1 Fig Determination of PHB content (expressed as % of cell dry weight (CDW)) in *C. necator* H16 during freezing-thawing treatment (-30°C).**


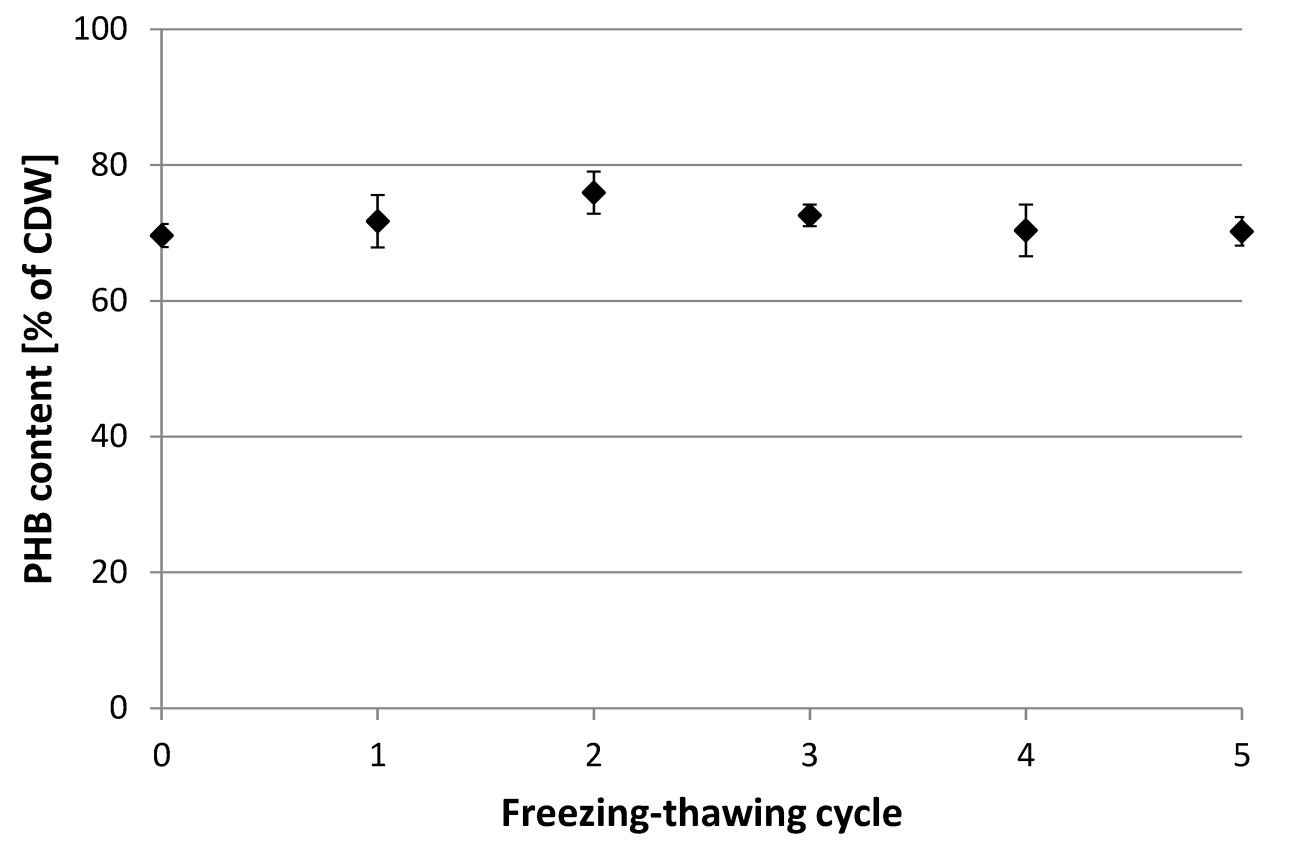

Supplement: S1 Fig — (DOCX) [file pone.0157778.s001.docx]
